# Disentangling covariate effects on single-cell-resolved epigenomes with DeepDive

**DOI:** 10.1016/j.patter.2026.101589

**Published:** 2026-06-17

**Authors:** Andreas Fønss Møller, Jesper Grud Skat Madsen

**Affiliations:** 1 Department of Biochemistry and Molecular Biology, University of Southern Denmark, 5230 Odense M, Denmark; 2 The Novo Nordisk Foundation Center for Genomic Mechanisms of Disease, Broad Institute of MIT and Harvard, Cambridge, MA 02142, USA

**Keywords:** single-cell epigenomics, disentanglement, representation learning, type 2 diabetes, single-nucleus ATAC-seq

## Abstract

Understanding the effects of individual biological factors from single-cell-resolved epigenomic data is hindered by multicollinearity, particularly in human cohorts. We introduce DeepDive, a deep-learning framework designed to systematically disentangle known and unknown sources of variation in single-nucleus ATAC-seq data. DeepDive accurately reconstructs chromatin accessibility, outperforms state-of-the-art methods with incomplete covariate information, and robustly recovers true biological signals from even highly entangled covariates, unlocking counterfactual, “what-if,” analyses. Applying DeepDive to pancreatic islet cells, we perform counterfactual analyses to prioritize covariates associated with a type 2 diabetes-linked beta-cell subtype and nominate transcription regulators. DeepDive offers a powerful and unbiased tool for mechanistic discovery in complex human disease cohorts.

## Highlights

• DeepDive models chromatin accessibility and covariates for counterfactual prediction
• DeepDive improves differential accessibility detection with confounded or missing data
• Applied to islets of Langerhans, DeepDive nominates regulators of type 2 diabetes

## The bigger picture

Single-cell genomics makes it possible to see how genes and the environment influence the behavior of individual cells in health and disease. In large-scale human cohort studies, however, key variables such as clinical traits (e.g., weight or age) and technical confounders are often correlated. This makes it difficult to tell which factors are actually driving changes seen in cells. DeepDive addresses this challenge by separating the effects of different patients, diseases, and technical factors in single-cell data. By separating these overlapping sources of variation, DeepDive helps identify regulatory changes linked to disease and improves the interpretation of complex cohort data.

## Teaser

Disentangling true biological signals from confounding factors remains a major challenge in single-cell genomics. Here, Møller and Madsen present DeepDive, a deep generative framework that separates covariate effects in single-cell chromatin accessibility data. DeepDive improves reconstruction accuracy, enables counterfactual predictions, and enhances the detection of differential accessibility under complex confounding. Applied to human pancreatic islets, it reveals disease-associated regulatory programs and links chromatin variation to genetic risk and beta-cell functional states.

## Introduction

The epigenome of a single cell is a fingerprint of its identity and a record of its exposure to environmental, developmental, and genetic factors that, via dynamic and complex interactions, shape cellular function and fate through transcriptional regulation. We can map accessible chromatin in single nuclei at scale using single-nucleus assay of transposase-accessible chromatin with sequencing (snATAC-seq). Chromatin accessibility can be used for identification of *cis*-regulatory elements and as a proxy for their activity. However, separating the effects of individual sources of variation on chromatin accessibility remains a difficult problem, especially in human cohort studies where multicollinearity between covariates is common. Multicollinearity naturally occurs between related biological factors; for example, it is well established that obesity is a strong risk factor for developing type 2 diabetes. Thus, among type 2 diabetics, the prevalence of obesity is higher than among non-diabetics. Using conventional statistical approaches, such as those implemented in widely adopted toolkits for snATAC-seq analysis, including Signac^1^ and ArchR,^2^ multicollinearity leads to poor and unstable estimates of the effect of each covariate. A few computational methods, such as Biolord^3^ and DrVI,^4^ exist for learning disentangled representations from single-cell genomics data; however, they either (1) are designed for single-cell RNA-sequencing datasets, (2) aim to disentangle latent factors but not covariates, or (3) fail to meaningfully capture latent variation. As a result, key biological signals can be obscured, complicating the interpretation of disease mechanisms and cellular states.

## Results and discussion

**Figure 1:**
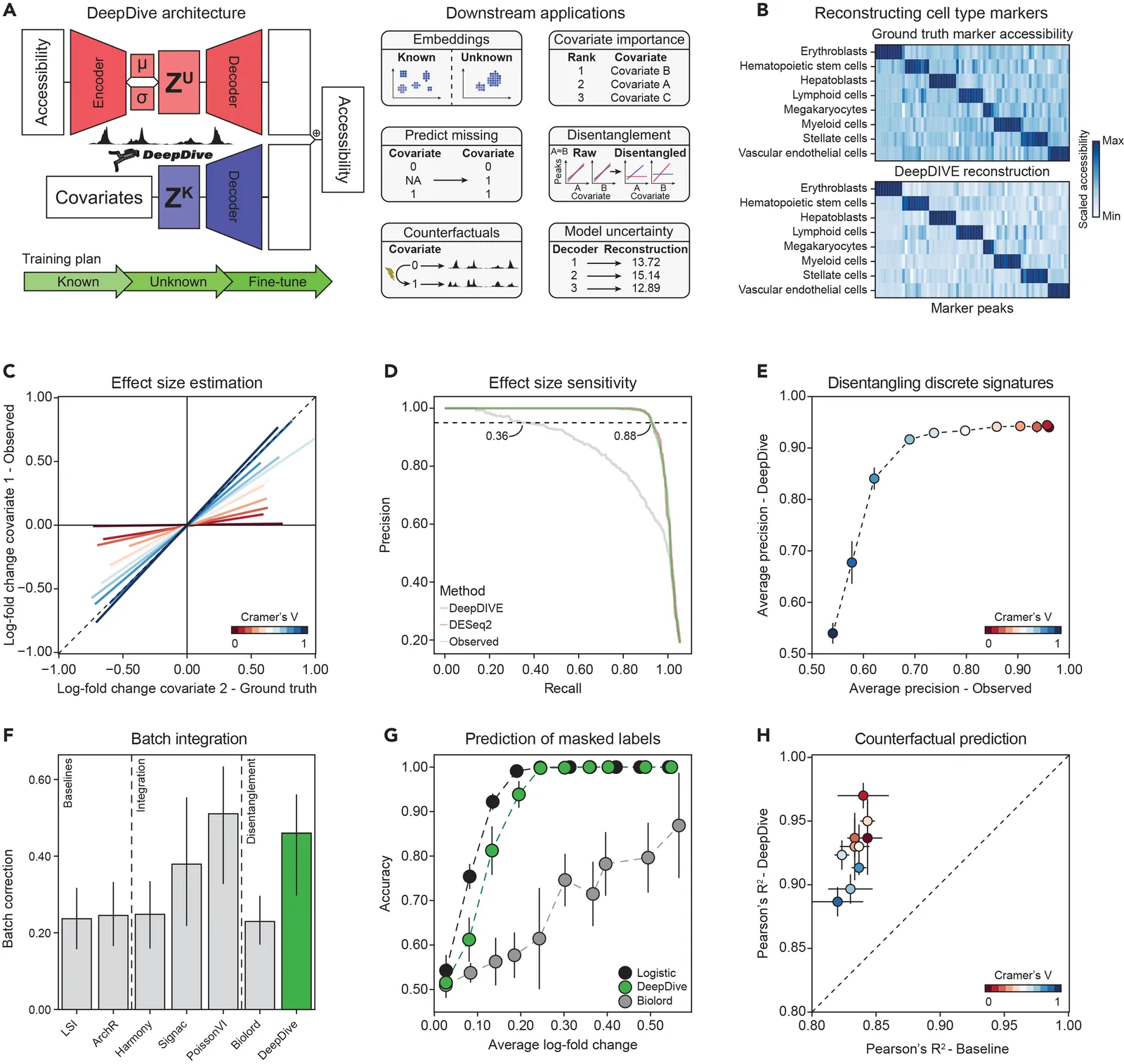
DeepDive accurately disentangles covariate effects on single-cell epigenomes (A) Schematic overview of the DeepDive model. The model consists of a dual-network architecture that disentangles known covariates using adversarial learning while modeling residual, unknown variation with a conditional variational autoencoder. (B) Heatmap showing pseudobulk chromatin accessibility at cell-type marker peaks in the raw dataset^6^ (top), and the DeepDive reconstruction after disentangling and removing donor-level covariates (bottom). (C) Lineplot showing signal leakage from a simulated covariate to another as a function of increasing multicollinearity strength. (D) Precision-recall curve comparing the performance in classifying differentially accessible peaks using DeepDive, DESeq2, or observed log_2_ fold changes. (E) Average precision for classifying differentially accessible peaks with discrete covariates, comparing DeepDive to log fold-change-based classification as a function of increasing multicollinearity strength. Error bars represent the standard deviation across three independent simulations. (F) Average batch correction metric across the five datasets. Error bars represent the 95% confidence interval of the mean (*n* = 5). “Baseline” indicates a standard LSI implementation. (G) Average accuracy of logistic regression (black), DeepDive (green), and Biolord (gray) predictions for masked covariate labels across effect sizes. Error bars represent standard deviations across three simulations. (H) Average Pearson’s *R*^2^ on hold-out covariate combinations. Error bars represent standard deviation.

To address this challenge, we introduce DeepDive, a computational framework for semi-supervised probabilistic modeling of sources of variation in single-cell-resolved epigenomic data for precise estimation of the effect of individual sources of variation, even among highly multicollinear factors. DeepDive is designed to be trained on individual cohorts in an intuitive and user-friendly manner. It is simple to train with just a few lines of code and has thorough documentation with guidance for training and interpretation. DeepDive consists of two networks (Figure 1A). The first network learns disentangled representations of known covariates using adversarial learning. The second network models the residual variation, which cannot be explained by known covariates, using a conditional variational autoencoder. This dual-network configuration separates known and unknown sources of variation and, therefore, enables the quantification of unknown variance and the identification of latent covariates. Utilizing a multi-decoder framework,^5^ DeepDive quantifies uncertainty in its own estimates, which can be directly used for differential accessibility testing. The model is trained end to end using a training scheme to maximize the information learned from known covariates. After training on the human liver dataset,^6^ predicted genome-wide and marker peak-specific chromatin accessibility is highly correlated with the observed signal. Furthermore, *de novo* recall of differentially accessible peaks using predicted counts showed a large overlap (average = 81.1%) with observed differential accessible peaks (Figures 1B and S1A–S1G). Collectively, these results indicate that DeepDive can accurately reconstruct shared and cell-type-specific chromatin accessible patterns.

In large-scale human studies, such as single-cell atlas generation, where datasets are integrated prospectively, covariates describing important biological factors may be partially or completely missing. For example, in the human lung cell atlas (HLCA), sex is missing for 47.5% of donors, age for 96.1% of donors, and smoking status for 32.6% of donors, and in the human retina cell atlas (HRCA), cause of death is missing for 80.8% of donors, while sex is missing for 7.7% of donors. To evaluate how imperfect information affects DeepDive, we trained DeepDive and Biolord, a state-of-the-art model for covariate disentanglement of covariates,^3^ on five different datasets with and without cell-type labels. We omitted cell-type labels because it is a strong covariate and, therefore, should be easy to separate in the basal latent space of each model, which captures residual variance. With cell-type labels, DeepDive and Biolord perform comparably, but in the absence of cell-type labels, reconstruction with DeepDive is significantly better correlated with observed signals for both cell-type-specific marker peaks (Figure S2A) and genome wide (Figure S2B), as DeepDive learns to separate cell types in basal latent space, while Biolord does not (Figure S2C).

In addition to imperfect information, single-cell atlases also often suffer from multicollinearity. For example, in the HLCA, there are varying degrees of multicollinearity, with smoking status as cause of death strongly collinear (Cramer’s *V* = 0.74), while sex is mildly collinear (Cramer’s *V* = 0.15). In the HRCA, there is also multicollinearity; for example, gender and age are collinear (Cramer’s *V* = 0.70). In a simulated dataset with no difference between groups, DeepDive showed well-calibrated test statistics with a slightly conservative behavior under the null (Figures S3A–S3G). To benchmark how well DeepDive can disentangle the effects of collinear covariates, we initially simulated datasets with various levels of collinearity with a known ground truth (Figures S4A and S4B). We confirmed that collinearity leads to poor effect-size estimates as the biological signal from one covariate “leaks” to the other (Figure 1C), thereby obscuring the true effects. At a moderate level of collinearity (Cramer’s *V* = 0.5) where all covariates are observed, we found that using DeepDive or simultaneously modeling both covariates with DESeq2^7^ improved recall to 88% for both approaches as compared to 36% for Wilcoxon rank-sum tests, at an accepted error rate of 5% (Figures 1D, S4C, and S4D). DeepDive and DESeq2 have a similar increase in performance relative to the number of donors (Figure S4E). When all covariates are not observed, we found that DeepDive maintains higher average precision (0.81) on features affected by the unobserved covariate compared to both DESeq2 (0.73) and Wilcoxon rank-sum (0.74) (Figure S4F). In fact, across all tested levels of collinearity, DeepDive consistently achieves similar or higher average precision for both discrete and continuous covariates compared to Wilcoxon rank sum (Figures 1E and S5A). This effect is dependent on the multi-decoder setup, and performance saturates at 20 decoders (Figures S5B and S5C). Of note, this performance comes at a trade-off in resources, as training time increases linearly with the number of decoders. The ability of DeepDive to accurately disentangle covariate effects is also robust to different levels of accessibility or missingness (Figures S5D and S5E; Table S1). Overall, these results indicate that DeepDive accurately and unbiasedly disentangles covariate effects. To test the ability of DeepDive to disentangle covariates in real data, we subset a human islet of Langerhans dataset^8^ to introduce collinearity between sex and diabetes status and compared effect-size estimates with the original dataset (Figure S5F); we found that DeepDive accurately estimates effect sizes, while Biolord performs worse than the observed fold change (Figure S5G).

The adversarial training of the known covariate network in DeepDive encourages the disentangled representations of observed covariates to be independent. This design enables counterfactual prediction, whereby the model is tasked with predicting chromatin accessibility for unseen covariate combinations. To test the accuracy of counterfactual prediction with DeepDive, we devised three tasks inspired by potential-use cases. In the first task, DeepDive was used for batch integration by omitting the cell-type variable during training and compared to Biolord and several methods specifically designed for integration using scIB.^9^ Although PoissonVI achieves the best overall integration performance, DeepDive attains comparable performance while additionally enabling explicit disentanglement of structured covariate effects and counterfactual inference. In contrast, Biolord achieves performance approximately on par with baseline methods (Figures 1F and S5H). In the second task, we use DeepDive for counterfactual prediction of missing donor covariates. To evaluate, we masked donor-level covariates with various effect sizes in simulated datasets and found that at moderate effect sizes, DeepDive predicts the missing covariate with high accuracy, closely matching the performance of a logistic regression model while additionally providing a unified framework for disentanglement and counterfactual inference (Figure 1G). In the same task, Biolord requires substantially larger effect sizes to achieve comparable predictive performance. Similarly, we found that DeepDive significantly outperforms baseline models in predicting diabetes status in real data (Figure S5I). In the third task, we use DeepDive to counterfactually predict chromatin accessibility of unseen combinations of covariates. To evaluate, we hold out a covariate combination from simulated datasets and compare counterfactual prediction to the hold-out ground truth. As an illustrative example, in a dataset with two cell types (A and B) and two donors (X and Y), we hold out cell type A in donor X, but A remains observed in donor Y. We evaluated DeepDive by virtually shifting the label of cell type B to A in donor X and comparing the predicted profile to the held-out data. We found that the counterfactual profile is significantly more similar to the ground truth than the baseline label (Figure 1H). To evaluate in real data, we held out a cell type in one donor and compared the counterfactual epigenome to the ground truth. We found that DeepDive accurately reconstructs the unseen epigenome more accurately than using the average profile of the same cell type from other donors (Figures S5J and S5K). In summary, DeepDive can disentangle collinear covariates for accurate reconstruction and counterfactual prediction of single-cell-resolved epigenomes.

**Figure 2:**
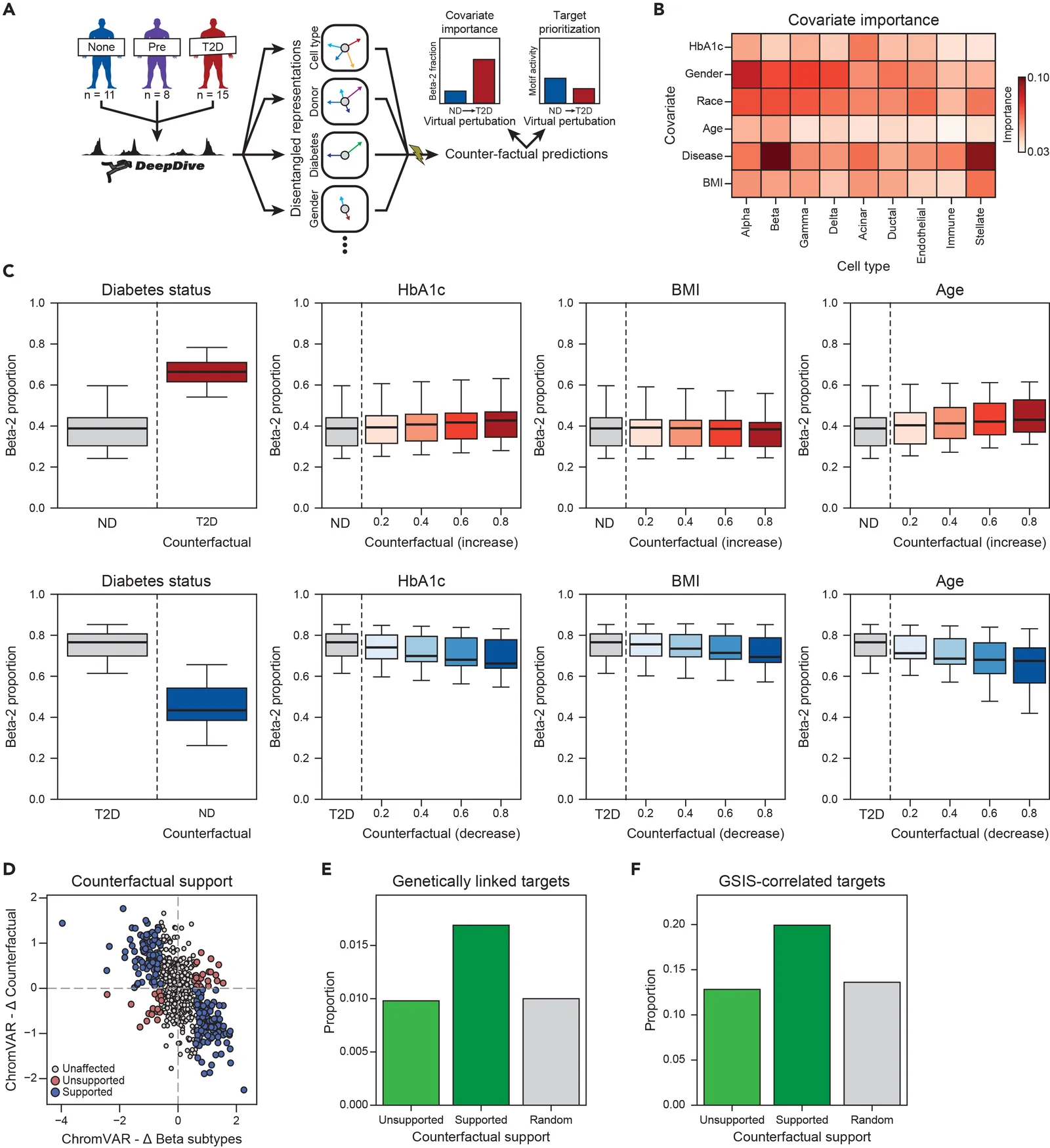
DeepDive disentangles covariate effects in pancreatic islet cells (A) Schematic overview. (B) Heatmap showing cell-type-specific covariate importance as determined by DeepDive. (C) Boxplots showing the predicted beta-2 cell proportion based on counterfactual prediction perturbing the indicated variables for non-diabetic (ND) (top) and type 2 diabetic (T2D) (bottom) individuals. (D) Scatterplot showing the change in ChromVAR motif activity between beta subtypes (*x* axis) as an effect of T2D to ND counterfactual prediction (*y* axis). (E and F) Barplots showing the proportion of transcription factor activities associated with beta-1 and beta-2 differences, stratified by DeepDive counterfactual support and random sampling, overlapping with eQTL and metabolic GWAS colocalization data (E) or GSIS correlated targets (F).

As a vignette of how counterfactual prediction with DeepDive can be used to nominate cell types and regulators linked to specific covariates, we trained a DeepDive model on snATAC-seq performed on human islets of Langerhans (Figure 2A) accounting for both biological covariates including cell type, disease status, body mass index (BMI), hemoglobin A1c (HbA1c), age, gender, and race, and technical covariates including center, storage, purity, and fraction of mapped reads (see Note S1). DeepDive accurately reconstructs the dataset from the known covariates, although these do not explain the entire epigenome (Figure S6A). We initially focused on the interaction between obesity (as proxied by BMI) and diabetes status, which is known to be entangled.^10^ To prioritize cell types affected by BMI and diabetes status, we used DeepDive to perform cell-type-resolved covariate importance and found that both covariates most strongly affect beta cells (Figure 2B). To validate that DeepDive has disentangled these covariates, we identified differential accessibility peaks associated with each covariate and found the two peak sets to be orthogonal (Jaccard index = 0.1, Figure S6B). Further, we leveraged human genetics and evaluated enrichment for genetic variants associated with type 2 diabetes and BMI. We found the peaks associated with BMI in DeepDive in beta cells are significantly enriched for variants linked to obesity but not for variants linked to type 2 diabetes (Figure S6C). Next, we set out to dissect how each donor-level covariate affects the proportion of the beta-1 and beta-2 cell subtypes, whose proportion has recently been shown to be strongly associated with HbA1c levels and diabetes status.^8^ We reconstructed chromatin accessibility without technical confounders and trained an XGBoost model to classify beta cells as beta-1 or beta-2, based on the authors original labels (Figures S6D–S6F). Next, we virtually shifted donor covariates and found that disease status has the strongest effect on the proportion of the two states, followed by small additive effects from HbA1c, BMI, and age (Figure 2C). To nominate transcription factors involved in the transition between these two beta-cell states, we used chromVAR^11^ to obtain motif activity estimates using the reconstructed chromatin accessibility profiles and using counterfactual profiles virtually shifting the diabetes status label. We identified 226 transcription factors associated with motifs differentially active between beta-1 and beta-2. Of those, the differential activities of 188 were rescued in counterfactual prediction (supported) and 38 were not (unsupported) (Figure 2D and Table S2). The list of supported transcription factors includes, for example, HNF1A, NKX6.1, PAX6, FOXA2, NeuroD1, HNF4A, and GLIS3, all of which are known regulators of beta-cell function.^12^ This enrichment of known regulators indicates that DeepDive nominates putative regulators with high accuracy. To add further support for the putative regulators, we leveraged both humane genetics and functional data and found that the regulators supported by counterfactual prediction were more enriched among proteins linked to type 2 diabetes through genetic evidence (Figure 2E) and correlated with glucose-stimulated insulin secretion (GSIS) (Figures 2F and S6G). Collectively, this strongly indicates that counterfactual prediction with DeepDive nominates high-confidence transcriptional regulators of significant interest for follow-up experiments.

DeepDive has limitations. It assumes that all covariates carry a signal, are partially independent, are separable in non-linear space, and are linearly additive in latent space. For example, hierarchical or nested covariates may violate independence assumptions, leading to representations where effects are not cleanly separated. In most cases, we expect these assumptions to hold when covariates represent independent processes. Thus, prior to modeling, covariates should be selected to avoid complete collinearity or redundancy, and after modeling, these assumptions should be evaluated, for example, through inspection of adversarial losses. We envision that future work can relax these limitations, for example, by using multiplicative rather than additive fusion, which would allow for non-linear interactions between known and unknown sources of variation. In conclusion, we present DeepDive, an end-to-end probabilistic framework capable of disentangling covariate effects on single-cell-resolved epigenomes, thereby enabling accurate counterfactual prediction.

## Methods

### DeepDive

DeepDive is a deep generative model designed to disentangle known and unknown sources of variation in high-dimensional count data from single-cell-resolved epigenomics assays. It combines a variational autoencoder (VAE) framework with adversarial networks to separate structured variation attributable to known covariates from residual, unannotated signals. The model specifics are described in greater detail in Note S1.

In DeepDive, observed single-cell-resolved profiles are modeled as arising from a latent representation that is partitioned into two components: (1) covariate-associated latent variables that capture structured effects of known sample- or cell-level annotations (e.g., donor phenotype or BMI), and (2) residual latent variables that capture remaining biological or technical variation. DeepDive employs an additive architecture to combine these components. Covariate-specific embeddings combine additively to form a single latent representation of known covariates. Predictions are then obtained through an additive decomposition at the output level, where decoded contributions from the known and unknown latent spaces are combined at the logit level. Conceptually, the addition of latent embeddings within the known branch enables DeepDive to model complex interactions between known covariates, while the addition of logits from the known and unknown branches provides stable decomposition and clear covariate attribution. This framework is essential for the utility of DeepDive and separates its ability to disentangle covariates from classical statistical approaches, as DeepDive automatically models unobserved sources of variation and models complex non-linear interactions without explicitly specifying high-order interaction terms. Adversarial classifiers and regressors are trained jointly with the VAE to minimize the predictive power of covariates in the residual latent space, ensuring effective disentanglement of annotated and unannotated effects. DeepDive estimates model uncertainty using a multi-decoder setup.^5^

The generative model reconstructs high-dimensional accessibility profiles directly from these disentangled representations, enabling both counterfactual prediction and downstream interpretability. Specifically, by holding the residual latent space fixed while manipulating covariate-associated latent space, DeepDive can simulate chromatin accessibility under hypothetical covariate settings, thereby estimating covariate-specific effects, which is not possible with simpler linear models.

### Model architecture and training procedure

DeepDive was implemented with two hidden layers of 128 and 64 fully connected nodes in both the encoders and decoders, and a latent space of 32 dimensions. Adversarial networks were trained with a single hidden layer of 16 fully connected nodes. Unless otherwise indicated, DeepDive was trained using 20 decoders. During training, DeepDive randomly selects one decoder to update for every minibatch. Dropout was applied at a rate of 0.1 in the autoencoder components and 0.3 in the adversarial networks. DeepDive is trained in three phases. In the first phase, only the known network is optimized in isolation. In the second phase, the known network is frozen, and the unknown (residual) network is optimized. In the last phase, the unknown network is frozen, and the known network is fine-tuned. The rationale for this three-step training plan is to promote maximization of the information explained by the known network and to separation of covariate effects from residual latent variation. The model was trained for 300 epochs per decoder, evenly distributed across the three phases. Optimization was performed using AdamW, with learning rates of 0.0001 for autoencoders and 0.0003 for adversaries and weight decay values of 0.001 and 0.0000004, respectively.

### Simplified workflow

DeepDive is designed to be trained on individual cohorts using single-cell chromatin accessibility counts and associated annotations. The required input is a cell-by-feature raw count matrix, e.g., fragment counts per peak and optional inputs include covariates, either sample-level (categorical or continuous, e.g., disease state or BMI) or cell-level (categorical or continuous, e.g., cell-type labels or quality metrics). To obtain a cell-by-feature count matrix, standard snATAC-seq preprocessing, including quality filtering, peak calling, quantification, and—optionally—feature selection, is necessary. Training is performed automatically in an end-to-end manner. Training time depends on model size and the number of cells and features and is substantially faster on a GPU than on a CPU. However, the model is lightweight and in most configurations can be trained on, for example, in MacBook M1 or better. After training, the users can perform conditional accessibility reconstruction, feature-level effect size estimation and test statistics, counterfactual prediction, prediction of missing covariates, and analysis of the residual latent representation capturing unknown variation. The documentation provides a more detailed workflow (https://deepdive-tutorial.readthedocs.io/).

### Effect-size estimation and differential analysis

To estimate differential effects attributable to a covariate, DeepDive leverages the multi-decoder framework to compared decoder reconstructions under different covariate embeddings while holding all other background covariates constant. Thus, effect sizes and confidence are estimated directly from the model by the ensemble of decoders, providing an empirical measure of uncertainty based on variability across independently trained model components. This contrasts with other approaches, where uncertainty estimates are derived from estimates across different cells. For discrete covariates, group-specific embeddings (e.g., group A vs. group B) were obtained from the model’s learned embedding space. For continuous covariates, the embedding was evaluated at the specified value and compared against a reference (zero). Where relevant, additional background covariates were fixed by adding their corresponding embeddings to the latent representation prior to reconstruction. For each covariate configuration, reconstructed feature values were obtained from all decoder networks. Feature-level differences between group A and group B reconstructions were computed per decoder. Within each decoder, a standardized test statistic was calculated by normalizing the difference across features to obtain a *Z* score, and a corresponding two-sided *p* value was derived from the standard normal distribution. To combine evidence across multiple decoders, *p* values for each feature were aggregated using Fisher’s method by default:

$${Χ}^{2}=-1\sum_{d=1}^{D}\ln\left(p_{d}\right)\text{,}$$

where *D* is the number of decoders. Optionally, *p* values can also be combined using Cauchy’s method, which may be less sensitive to extremely small *p* values and offer more robustness for correlated *p* values. Effect sizes were summarized as the mean reconstructed difference across decoders. Multiple testing correction was applied to combined *p* values across all features using the Benjamini-Hochberg false discovery rate (FDR) procedure.

### Evaluation of effect-size estimates

To evaluate the differential effect-size estimation procedure, a simulation of single-nucleus chromatin accessibility profiles under controlled multicollinearity between discrete (Cramer’s *V* 0–1) and continuous (Pearson correlations 0–1) covariates was utilized. The presence of a non-zero simulated effect was the ground truth.

Estimates were likewise evaluated on beta cells from a real scATAC-seq data of pancreatic islets^8^ downsampled (20,000 cells) to induce multicollinearity between disease status and sex (Cramer’s *V* 0–1). Ground-truth differentially accessible peaks were identified in rebalanced data (40,000 cells, Cramer’s *V* ∼0).

Performance was quantified using average precision from the precision-recall curve. DeepDive-predicted effect sizes were compared to log fold changes calculated on the simulated and downsampled data. Effect-size estimation with log fold change and Wilcoxon rank-sum differential accessibility testing was performed with scanpy^13^ rank_genes_groups with method = wilcoxon on log-transformed counts.

### Simulating count data with controllable covariate dependence

A simple simulation framework was implemented to generate single-nucleus chromatin accessibility profiles with controlled covariate influence. The simulation produces fragment counts with a specified number of cells and genomic features (peaks) while allowing covariates to modulate accessibility.

Each dataset $X\in N^{n\times m}$ is initialized with baseline accessibility values drawn from a uniform distribution across features *μ*_*j*_ ∼ Uniform(0.5, 2.0), *j* = 1, …, *m*. Cells are then assigned to covariate groups with probability *P*. If assuming dependency between two covariates, these are drawn from a joint probability distribution $P\in R^{r\times c}$ specifying the relationship, where *r* and *c* are the number of groups in the two covariates. To get this distribution only parameterized by an entanglement score *η* ∈ [0, 1], where 0 is independence and 1 is perfect dependence, *P*(*η*) = (1 − *η*)*U* + *η*Ĩ, where *U* is a uniform joint distribution *U*_*ij*_ = 1/*rc* and Ĩ = 1/min(*r*, *c*)pad(*I*_min⁡(*r*,*c*)_, thereby interpolating between perfect independence and dependence. Covariates are assumed to have independent effects, and, thus, feature-level effect sizes are introduced by randomly selecting a subset of features for each covariate with proportion QC and applying multiplicative adjustments to their baseline accessibility. For each covariate *c* and group *g*, an effect vector is defined as $\alpha_{j}^{\left(c,g\right)}∼\text{ScaledNormal}\left(1,\sigma^{2};1-\delta_{c},1+\delta_{c}\right)$, where *δ* is the maximum effect size applied to selected features and $\alpha_{j}^{\left(c,g\right)}=1$ to non-selected features. For cell *i*, the mean accessibility of feature *j* is therefore given by $\lambda_{ij}=\mu_{j}\prod_{c}\alpha_{j}^{\left(c,g_{i}^{c}\right)}$. The final counts are finally sampled from a Poisson distribution with mean values after adjustment *X*_*ij*_ ∼ Poisson(*λ*_*ij*_).

### Benchmarking of integration methods

Integration performance was benchmarked using the scIB framework.^9^ We compared four methods: latent semantic indexing (LSI, uncorrected), ArchR^2^ integrative LSI, Harmony,^14^ Signac^1^ reciprocal LSI, PoissonVI,^15^ and DeepDive. The methods were applied to five independent publicly available scATAC-seq datasets^6^^,^^8^^,^^16^^,^^17^^,^^18^ spanning 27,034–720,613 cells and 5–34 donors.

Benchmarking was conducted using scIB,^9^ which evaluates integration along two complementary axes: biological conservation and batch correction. Biological conservation was quantified using metrics of cell-type separation and neighborhood preservation, including normalized mutual information (NMI) and adjusted Rand index (ARI) with Leiden clustering, silhouette width with respect to cell-type annotations, and the cell-type local inverse Simpson’s index (cLISI). Batch correction performance was assessed using the integration LISI (iLISI) for batch mixing, graph connectivity, and additional global measures of batch effect removal.

### Datasets and preprocessing

We used the following datasets and covariates.

(1) NeuroIPS^17^ (GEO: GSE194122): Cell type, batch, site, DonorID, DonorBloodType, DonorRace, Ethnicity, DonorGender, DonorSmoker, DonorAge and DonorBMI.
(2) Kidney^16^ (GEO: GSE151302): Celltype, sample. Age, gender, race, and egfr.
(3) GCC^18^ (GEO: GSE162170): Iterative.LSI.Clusters, Sample.ID, Age, Tissue.ID, Sample.Type, Batch, and Age.
(4) sciATAC-seq3^6^ (GEO: GSE149683): Cell_type, donor_id, sex, batch, tissue, and day_of_pregnancy.
(5) Pancreas^8^ (GEO: GSE169453): Cell_type, donor, Diabetes status, Human islet resource center, sex, ethnicity, 10× multi-ome assay, islet index, age, BMI, HbA1c, and purity.

In benchmarking all datasets, peaks accessible in 10% of cells and cells with more than five accessible peaks were retained for analysis. In the vignette of disentangling the effects to diabetes status and obesity, the thresholds were relaxed to peaks accessible in 1% of cells. To approximate fragment counts, we rounded uneven counts to the next highest even number and halved the count.^15^

### Disentanglement with Biolord

Biolord^3^ was applied to the single-cell-resolved accessibility data to learn disentangled representations of biological and technical covariates. The model was run following the authors’ recommended parameters as described in the official vignette.

### Counterfactual prediction of missing covariate combinations

To evaluate whether DeepDive can generalize to unseen combinations of covariates, a controlled simulation study was performed. Single-cell-resolved chromatin accessibility profiles were simulated under two binary conditions with varying multicollinearity (Cramer’s *V* 0–1). To assess counterfactual prediction, all cells corresponding to one covariate combination were removed from the training data. A DeepDive model was trained on the remaining cells. The trained model was subsequently used to reconstruct chromatin accessibility profiles for (1) cells from an observed covariate state and (2) a counterfactual prediction with a covariate switched to the unobserved covariate. Predicted accessibility profiles were compared against the true profiles that were held out and evaluated for non-zero effect-size features. Model accuracy was quantified by computing the Pearson coefficient of determination (*R*^2^) between the mean predicted and ground-truth accessibility.

### Predicting missing covariates

#### Cell-level covariates

DeepDive’s counterfactual reconstruction was used to impute missing cell-level covariates. For each covariate with missing values, we ranked its relative importance to define an inference order. Candidate values were taken as all observed categories for discrete covariates and as evenly spaced samples across the observed range for continuous covariates. Cells with missing values were reconstructed under each candidate while holding other covariates fixed. Reconstruction accuracy was assessed using the negative log likelihood between reconstructed and observed accessibility profiles, and the candidate minimizing this error was assigned as the predicted covariate.

#### Donor-level covariates

For donor-level inference, we masked the diabetes status of a single donor and trained DeepDive while excluding donor-specific variation. We then generated non-diabetic (ND) and type 2 diabetic (T2D) counterfactual reconstructions for all unmasked donors. Differences between ND and T2D reconstructions, together with cell-type labels, were used to train an XGBoost classifier. For the masked donor, predictions were made on its counterfactual differences, and cells with class probabilities ≥0.975 contributed to a majority vote used to assign the donor label.

### Linkage disequilibrium score regression

DeepDive was applied to identify peaks uniquely associated with BMI and type 2 diabetes (FDR < 0.05, effect size >0.1). Peaks were lifted over from hg38 to hg19 and overlapped with 1000 Genomes Project Phase 3 EUR SNPs within a 1,000 bp window centered on each peak. Partitioned heritability was estimated using stratified linkage disequilibrium score regression (S-LDSC)^19^ with the baselineLD v.2.2 model.

### Counterfactual support

For motif activity analysis, pyChromVAR^11^ was used to estimate motif activity of clustered non-redundant motifs^20^ on reconstructed profiles and T2D-to-ND counterfactuals, adjusting for the covariates cell type, disease, sex, race, age, BMI, and HbA1c, with default parameters. Motifs showing an absolute difference in chromVAR deviation >1 between beta-1 and beta-2 cells were classified as regulated, while those below this threshold were considered unregulated. Regulated motifs were further stratified by direction of change in T2D-to-ND counterfactuals: motifs reversing the beta-subtype effect were designated “supported,” whereas those with the same directional effect were designated “unsupported.” Each supported and unsupported motif is associated with multiple transcription factors that can bind to that motif. We extracted and tested all associated transcription factors for enrichment among factors correlated with GSIS or with genetic evidence.

GSIS-associated transcription factors were defined by per-gene ordinary least-squares regression, modeling the relationship between gene expression and the GSIS stimulation index while adjusting for culture time, donor age, donor sex, cold ischemia time, islet purity, cell-trapping percentage, and digestion time (FDR < 0.01; expressed in ≥1% of pancreatic islet cells^8^).

Transcription factors with genetic support were obtained from a database of expression quantitative trait loci (eQTLs) colocalizing with genome-wide association study (GWAS) loci for metabolic traits,^21^ restricted to transcription factors expressed in ≥1% of pancreatic islet cells.^8^

### Statistics and reproducibility

The statistical analysis of the data was as described in the aforementioned methods and can be reproduced from scripts available on GitHub (http://github.com/madsen-lab/DeepDive_reproducibility). No statistical tests were used to predetermine sample size. When simulating or downsampling datasets, the process was repeated three times, and average scores were reported. The investigators were not blinded to allocation during experiments and outcome assessment.

## Resource availability

### Lead contact

Requests for further information and resources should be directed to and will be fulfilled by the lead contact, Jesper Grud Skat Madsen (jgsm@bmb.sdu.dk).

### Materials availability

This study did not generate new unique reagents.

### Data and code availability

All datasets analyzed or used for benchmarking are publicly available and summarized in the datasets and preprocessing section. DeepDive is available as a Python package at http://github.com/madsen-lab/DeepDive. The code to reproduce the results is available at http://github.com/madsen-lab/DeepDive_reproducibility. The code has similarly been deposited at Zenodo (https://zenodo.org/records/19690489).^22^

## Acknowledgments

This work was supported by grants from the 10.13039/501100009708Novo Nordisk Foundation (NNF21SA0072102 and NNF21OC0068929, J.G.S.M.) as well as the 10.13039/501100001732Danish National Research Foundation (DNRF141, J.G.S.M.) to the Center for Functional Genomics and Tissue Plasticity (10.13039/501100014713ATLAS). Computation for this project was performed using the UCloud interactive HPC system, which is managed by the eScience Center at the University of Southern Denmark.

## Author contributions

A.F.M. and J.G.S.M. conceptualized the method; A.F.M and J.G.S.M. implemented the code, performed analysis, and prepared the figures; J.G.S.M. supervised the work; J.G.S.M. wrote the manuscript with input from A.F.M.; and A.F.M and J.G.S.M. contributed to manuscript editing.

## Declaration of interests

The authors declare no competing interests.

## Declaration of generative AI and AI-assisted technologies in the writing process

During the preparation of this work, the authors used ChatGPT for language editing. After using this service, the authors reviewed and edited the content as needed and take full responsibility for the content of the published article.

## Supplemental information

Document S1. Figures S1–S6 and Note S1

Table S1. scIB batch integration metrics Batch integration metrics across five datasets and seven methods.

Table S2. Counterfactual support for transcription factor motifs Transcription factor motif activity differences between beta-1 and beta-2 subtypes and diabetes status counterfactual support.

Table S3. Simulation parameters Description of simulation parameters.

Document S2. Article plus supplemental information
