# Supplementary material for: Disentangling covariate effects on single-cell-resolved epigenomes with DeepDive
Source: Patterns (N Y). 2026 Jun 17;7(9):101589. doi: 10.1016/j.patter.2026.101589 (PMC13576652; doi:10.1016/j.patter.2026.101589)
Supplement: Supplementary file 1 [file mmc1.pdf]

Patterns, Volume *7*

Supplemental information

Disentangling covariate effects on single-cell-resolved epigenomes with DeepDive

Andreas Fønss Møller and Jesper Grud Skat Madsen

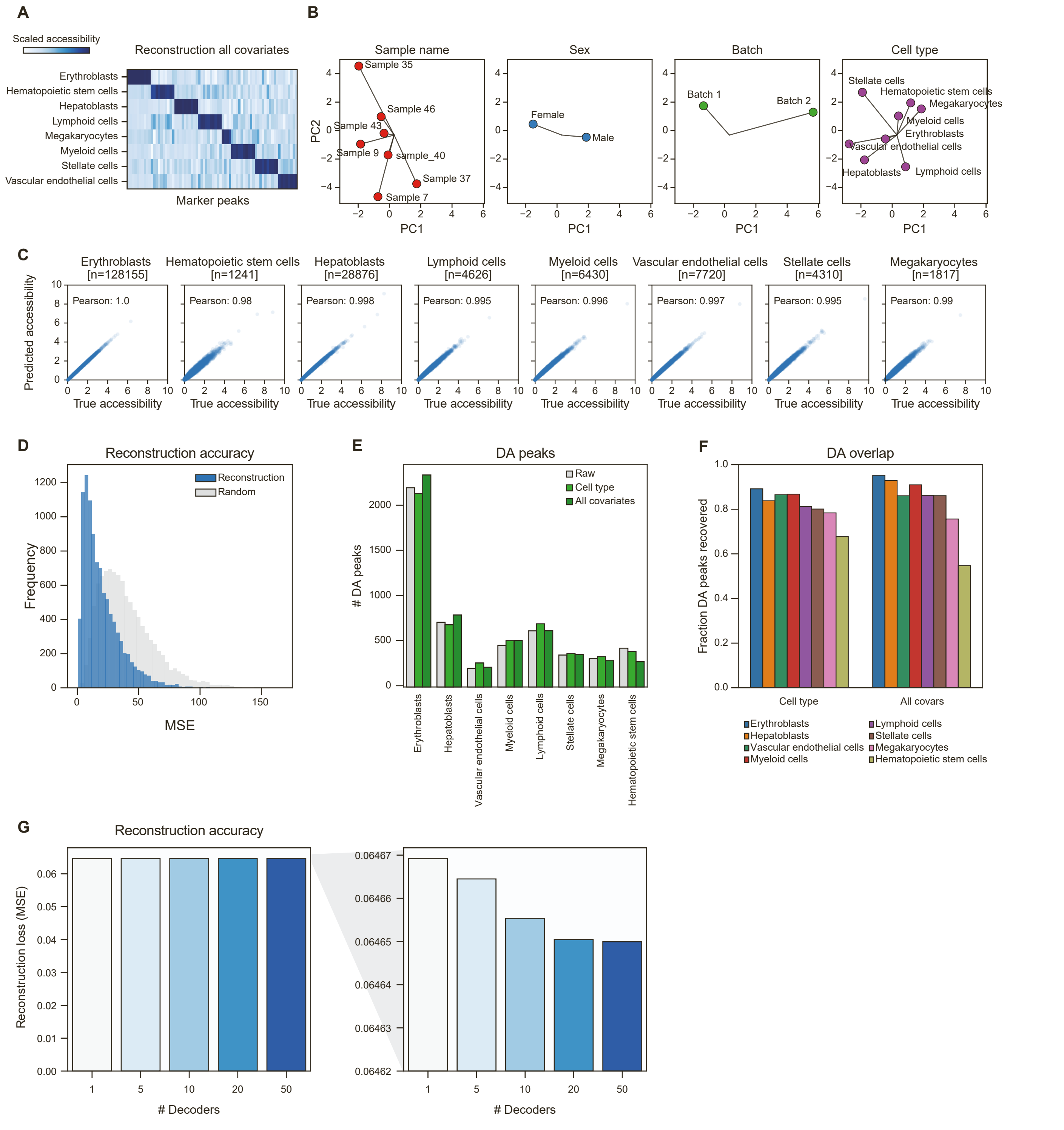

Figure S1: DeepDive accurately reconstructs cell type–specific chromatin accessibility

A) Heatmap showing pseudobulk chromatin accessibility at cell type marker peaks in the DeepDive reconstruction using all covariates. **B)** Scat-terplots showing the first and second principal components of the indicated covariate embeddings for known covariates. **C)** Scatterplots showing observed and DeepDive predicted average chromatin accessibility for the indicates cell types. **D)** Histogram showing the mean squared error (MSE) between the observed and DeepDive predicted chromatin accessibility and between the observed chromatin accessibility for random cell pairs. **E)** Barplot showing the number of marker peaks identified from the raw data and the DeepDive reconstructions. **F)** Barplot showing the frac-tion of marker peaks identified from the raw data that are successfully recovered in the DeepDive reconstruction. Reconstruction with only cell type labels on the left and reconstruction with all covariates on the right. **G)** Barplot showing the MSE of between observed and predict chromatin acces-sibility for DeepDive models trained with 1, 5, 10, 20, and 50 decoders.

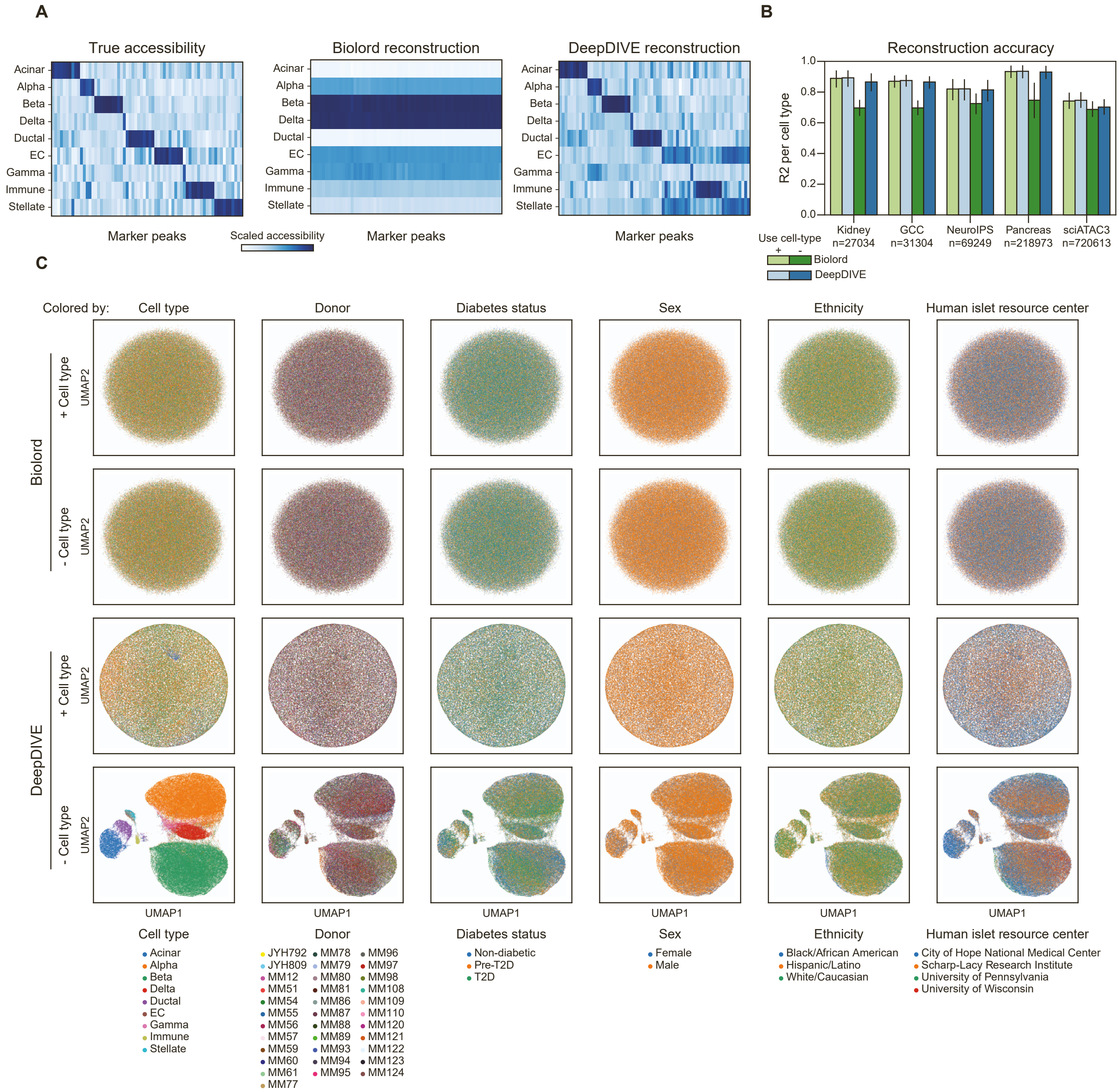

Figure S2: DeepDive captures biologically meaningful latent variation in the unknown embedding
A) Heatmap showing pseudobulk chromatin accessibility at cell type marker peaks from the human pancreas raw dataset (left) compared to reconstructions by Biolord (middle) and DeepDive (right). Both models were trained without explicit cell type information. **B)** Barplot showing average genome-wide reconstruction accuracy (R2) of cell type pseudobulk profiles (reconstructed versus ground truth), comparing Biolord and DeepDive models trained both with and without cell type information, across the five benchmarking datasets. Error bars represent the 95% confidence interval. **C)** UMAP visualizations of unknown variation embeddings from the pancreas dataset, generated by Biolord (top two rows) and DeepDive (bottom two rows). The top row for each model shows training with cell type information, while the bottom row shows training without.

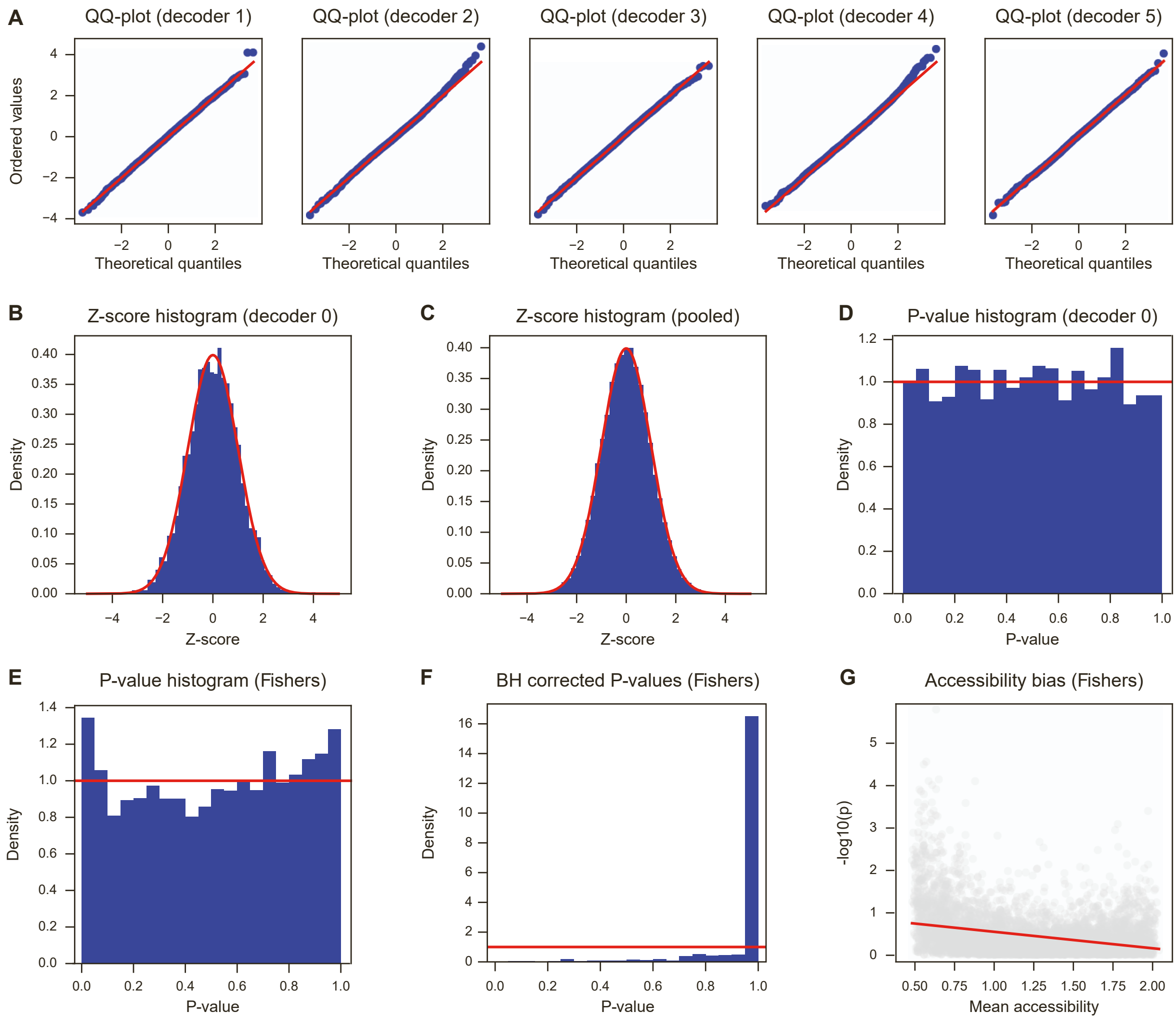

Figure S3: Statistical calibration and aggregation of decoder-derived Z-scores and P-values in DeepDive

A) QQ-plot of Z-scores for the first 5 decoders. **B-C)** Histograms showing Z-scores for the first decoder (B) and all decoders pooled (C). The red line indicates a Gaussian probability density function with mean 0 and standard deviation 1. **D-F)** Histograms showing of P-values of the first decoder (D), P-values aggregated across all decoders with Fisher's method (E), and Fisher's method aggregated P-values corrected with Benjamini-Hochberg (F). **G)** Scatterplot sowing the relationship between the per-feature Fisher's aggregated P-values and mean accessibility.

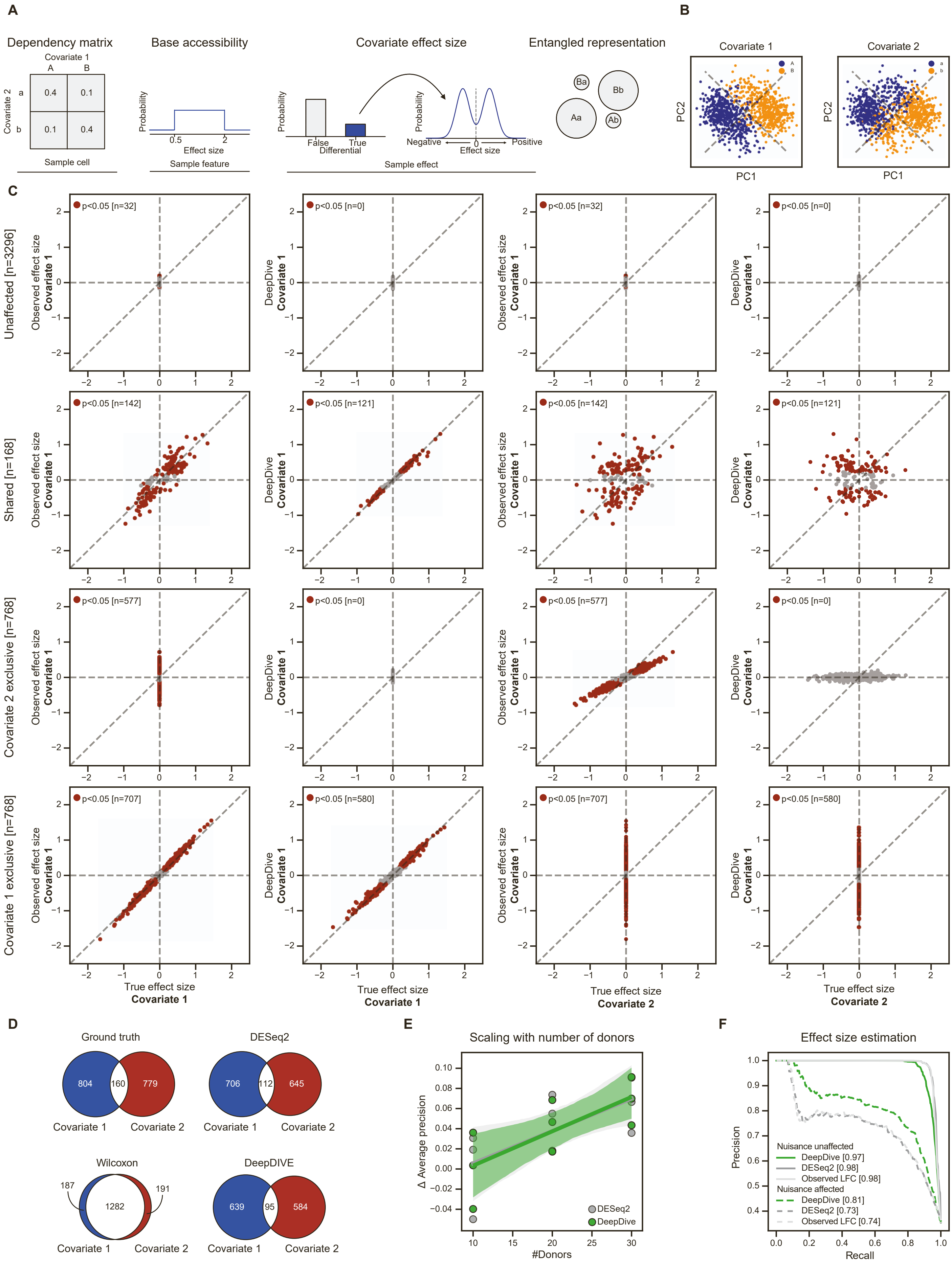

Figure S4: DeepDive robustly disentangles covariate effects under multicollinearity in simulated scATAC-seq data
A) Schematic illustrating the process of simulating scATAC-seq data with controlled multicollinearity between covariates. **B)** UMAP visualizations of the simulated dataset, displaying the distributions according to two entangled covariates with a Cramer's V of 0.5. **C)** Scatterplots showing accuracy of the estimated effect size for two entangled covariates, comparing estimates derived from log fold change calculations and hypothesis testing using Wilcoxon rank sum versus DeepDive. **D)** Venn diagrams comparing simulated differentially accessible peaks, showing ground truth, Wilcoxon, DESeq2 and DeepDive (left to right) under multicollinear conditions. **E)** Scatterplot showing change in average precision as a function of the number of donors. **F)** Line plots showing precision recall curve for features affected or unaffected by a nuisance covariate. Curve labels are annotated with the associated average precision scores.

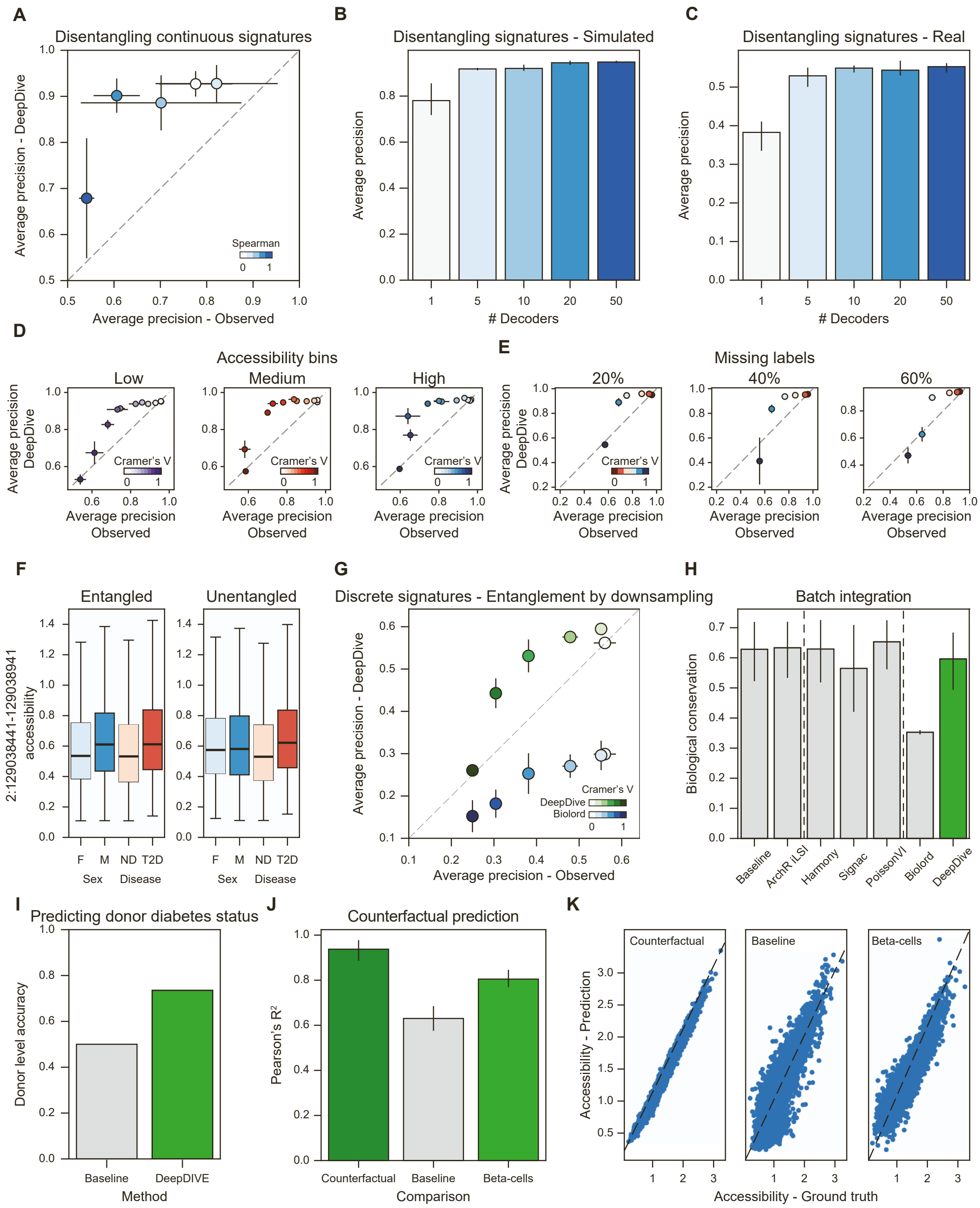

Figure S5: DeepDive enhances differential accessibility detection and robustness to multicollinearity

A) Scatterplot showing average precision for classifying differentially accessible peaks with continuous covariates, comparing DeepDive to log fold change-based classification (by grouping covariate values as high and low), as a function of increasing multicollinearity strength. **B)** Barplots showing the average precision for classifying differentially accessible (DA) peaks at a Cramer's V of 0.6 with 1, 5, 10, 20, and 50 decoders, in simulated data. **C)** Barplot showing the average precision for classifying DA peaks at a Cramer's V of 0.6 between disease status and sex, with 1, 5, 10, 20, and 50 decoders, achieved by downsampling the pancreas dataset5. **D-E)** Scatterplots showing average precision for classifying differentially accessible peaks comparing DeepDive to log fold change-based classification for discrete covariates across different accessibility bins (low, medium, and high) (D) and levels of missingness (20%, 40%, and 60%) (E). **F-G)** Classifying differentially accessible peaks with discrete covariates, comparing DeepDive to log fold change-based classification, as a function of increasing multicollinearity strength. The pancreas dataset was downsampled to induce multicollinearity between females (F) and males (M) and non-diabetics (ND) and type 2 diabetics (T2D) (Cramer's V 0-1). Illustrated is an example peak with and without induced entanglement (Cramer's V 0.5) (F) and average precision across multicollinearity strength (Cramer's V 0-1) for DeepDive (green) and Biolord (Blue) (G). **H)** Barplot showing the average biological conservation metric across the five datasets. Error bars represent the 95% confidence interval (n=5). Baseline indicated a standard LSI implementation. **I)** Barplot showing accuracy of predicting donor diabetes status using DeepDive versus baseline accuracy with XGBoost. **J-K)** Pearson R^2 of counterfactual, a baseline of acinar cells and beta cells from another donor compared to the hold-out donor beta cells (J) and selected example (K).

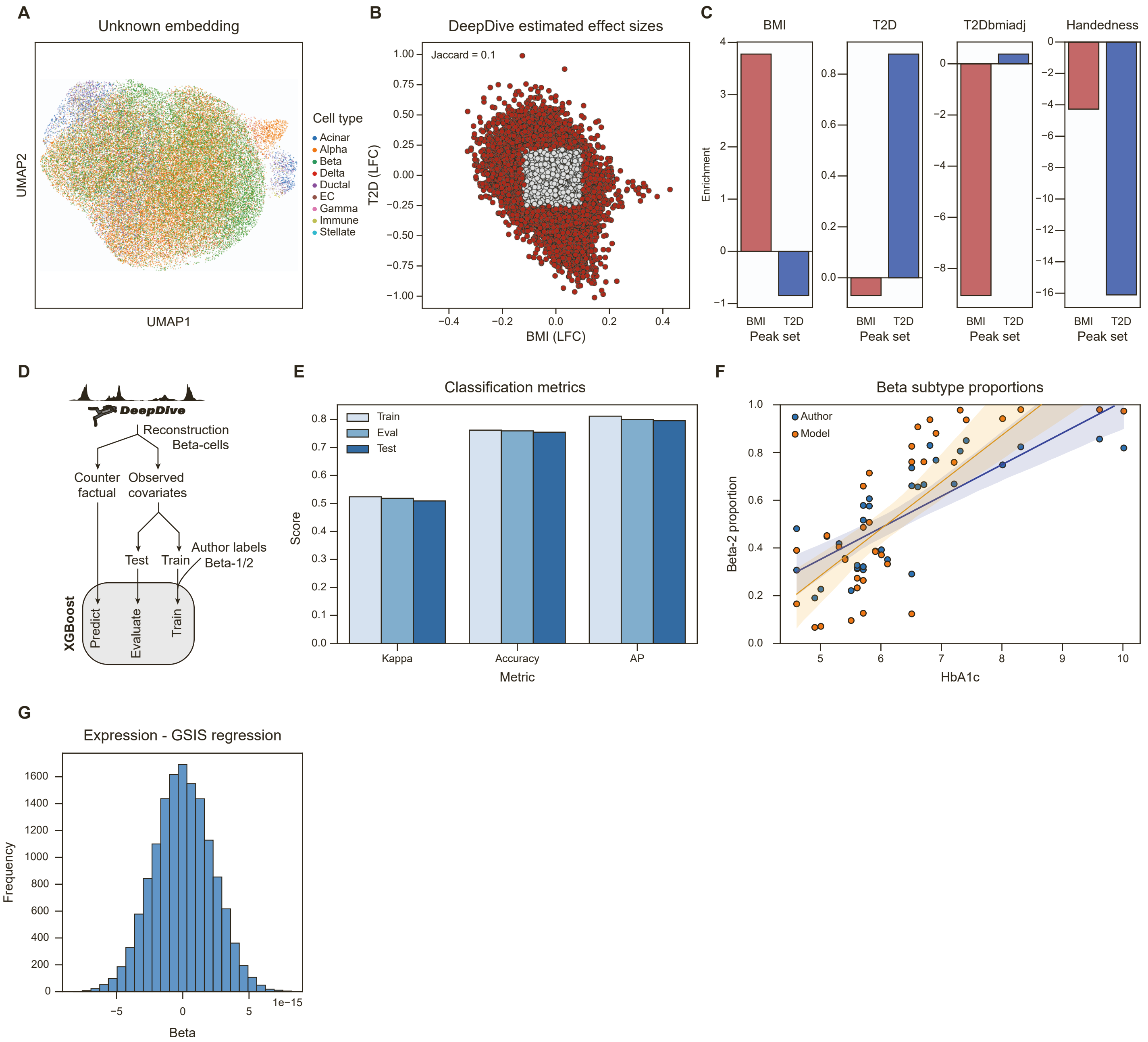

Figure S6: DeepDive links latent regulatory variation to disease genetics and beta-cell functional states
A) UMAP visualization of the basal embedding from DeepDive superimposed with cell type labels. **B)** Scatterplot showing DeepDive estimated effect sizes of BMI (x-axis) and diabetes status (y-axis) per peak. **C)** Barplot showing log2 enrichment of S-LDSC partitioned heritability in BMI and diabetes status associated peak sets for UKBB BMI, T2D, T2Dbmiadj, and handedness GWAS traits. **D)** Schematic of XGBoost beta-cell state prediction. **E)** Barplot showing scores for the XGBoost beta-cell state classifier for the train, validation, and test sets. **F)** Scatterplot showing beta-2 proportion against the HbA1c level for author and XGBoost labels. **G)** Histogram showing the regression coefficients for expression level against GSIS for each gene.

Supplemental Note 1

DeepDive is a deep generative model designed to disentangle known and unknown sources of variation in high-dimensional count data, from single-cell assays. It combines a variational autoencoder (VAE) framework with adversarial regularization to separate structured variation attributable to known covariates from residual, unannotated signals.

Generative model

The generative model assumes that single-cell genomics data can be described as the sum of contributions from known and unknown sources in latent space, where the known contribution is derived from structured covariate information and the unknown contribution is derived from the observed high-dimensional count vector x (e.g., chromatin accessibility). These contributions are modelled using two neural networks f_{known} and $f_{unknown}$.

The generative distribution over x given a latent space (z) is modeled as a Poisson distribution:

$$p(x|z) = \text{Poisson}(\lambda), \text{ where } \lambda = \exp(l)\,\text{Softmax}(f_{total}).$$

Where, $l \in \mathbb{R}$ is the log-library size factor, and f_{total} represents the additive contributions from known (c) and unknown (z) components where f() is a decoder:

$$f_{total} = f_{known}(c) + f_{unknown}(z)$$

Model Components

Unknown network: Let $x \in \mathbb{R}^D$ represent the observed high-dimensional count vector x (e.g., chromatin accessibility). We assume a latent variable $z \in \mathbb{R}^d$, drawn from a standard normal prior:

$$p(z) = N(z; 0, 1)$$

The latent variable z is inferred from the data using a neural network encoder:

$$q(z|x) = N(z; \mu(x), diag(\sigma^2(x)))$$

Where $\mu(x)$ and $\sigma(x)$ are parameterized by a neural network. We employ the reparameterization trick to allow for backpropagation through stochastic sampling:

$$z = \mu(x) + \sigma(x) \odot \epsilon, \epsilon \sim N(0,1)$$

The sampled latent variable z is passed through a decoder network $f_{unknown}$, producing the unknown contribution to the reconstruction:

$$\hat{x}_{unknown} = f_{unknown}(z)$$

Known network: The known network f_{known} maps structured covariates c (e.g., batch, cell-type, or condition labels) to a feature vector contributing to the reconstruction by decoding from covariate embeddings. The known decoder accepts categorical and continuous covariates, embedding them appropriately before transformation. Continuous covariates are further min-max scaled such that $C_j \in [0, 1]$.

For covariates, DeepDive uses trainable embedding matrices, where the input $C_j \in \{1, \ldots, k_j\}$ is represented as:

$$e_j = E_j[c_j], E_j \in \mathbb{R}^{K_j \times \mathrm{d}}$$

Continuous covariates are embedded as a covariate with $K = 1$. To improve generalization and robustness, Gaussian noise is added to the embeddings during training.

$$\tilde{e}_j = e_j + \epsilon, \epsilon \sim N(0, \sigma^2 I)$$

A sample with structured covariates $c = \{c_1, \ldots, c_j\}$, each is embedded as described above. The full covariate representation is fed to the decoder f_{known}as the sum of the covariate embeddings:

$$\hat{x}_{known} = f_{known}(\sum_{j=1}^{J} \tilde{e}_j c_{count})$$

Where c_{count} is the weight for continuous covariates that scales the embedding vector proportional to the continuous covariate magnitude. No scaling is applied to discrete covariates, effectively making $c_{count} = 1$.

Objective

DeepDive minimizes the standard evidence lower bound (ELBO), augmented with adversarial losses to encourage disentanglement:

$$\mathcal{L} = \mathbb{E}_{q(z|x)}[-\log(p(x|z)] + \beta \cdot KL(q(z|x) \,||p(z) + \mathcal{L}_{k\ adv} + \mathcal{L}_{uk\ adv}$$

The adversarial losses $\mathcal{L}_{adv}$ are described below.

Reconstruction loss: Since the output is modeled as a Poisson distribution, the negative log-likelihood is:

$$\mathcal{L}_{recon} = -\sum_{i=1}^{D} (x_i \log \lambda_i - \lambda_i - \log(x_i!))$$

Where, λ_i is the expected count for the feature i, derived from the sum of $\hat{x}_{known}$ and $\hat{x}_{unknown}$.

KL divergence: Encourages the posterior $q(z|x)$ to stay close to the Gaussian prior $p(z)$.

Adversarial Disentanglement

To encourage independence between known covariates and the learned latent space z, DeepDive employs adversarial predictors trained to infer each covariate from both the known and unknown components. These predictors are trained to minimize the task-specific error, while the generative model is trained to maximize it. Formally, for each covariate cj, the adversarial losses are:

$$\mathcal{L}_{k\ adv} = \sum_{j} \epsilon_j\big(g_j(s_j), c_j\big), \mathcal{L}_{uk\ adv} = \sum_{j} \epsilon_j\big(g_j(z), c_j\big)$$

$$s_j = \sum_{i \neq j}^{J} \tilde{e}_i c_{count}$$

Where g_j is the adversarial predictors for the covariate c_j, $\epsilon_j$is the covariate-specific error function and, s_j is the sum of the covariate embedding vectors with the embedding of j held out. For discrete covariates, j is the cross-entropy loss $-\sum_{k=1}^{K} c_i \log g_i^k(h)$ of Kclasses, and for continuous covariates, ϵ_j is the mean squared error loss $\big(g_i(h) - c_j\big)^2$, where $h \in \{s_j, z\}$.

Where each g_j is a neural network classifier (or regressor) predicting the covariate c_j. The gradient from $\mathcal{L}_{adv}$ is reversed during backpropagation to prevent known and unknown features from encoding redundant information about the covariates.

Optimization and scheduling

Optimization is performed using AdamW, with learning rate scheduling using ReduceLROnPlateau from PyTorch, based on the Poisson reconstruction loss. The model is optimized in different phases. In the first phase, only the known network is trained and affects reconstruction such that $f_{unknown(z)} = 0$ in the second phase, the known network weights are frozen, while the unknown network is fit. Finally, the known network is fine-tuned with the weights of the unknown network being frozen. This scheduling strategy is employed to discourage the unknown network from learning what is already captured by the unknown network.

Multi-decoder architecture for statistical inference

DeepDive allows instantiating **multiple parallel decoders** for both the known (f_{known}) and unknown ($f_{unknown}$) generative components. Rather than learning a single deterministic mapping from covariates or latent variables to feature space, DeepDive maintains a set of D decoder instances per component:

$$\{f_{known}^{d}\}_{d=1}^{D}, \{f_{unknown}^{d}\}_{d=1}^{D}$$

During training, decoders are updated independently, and during inference, any decoder can be selected for generating predictions. This design has two main advantages:

Model uncertainty and effect size variability: The decoder ensemble effectively captures the mapping variability between inputs and outputs. This allows users to quantify uncertainty over the predictions by computing, for example, the variance of feature-level predictions across decoders for the same input. Similarly, the differences in predictions across decoder instances can be used to derive posterior-like distributions over effect sizes.

For a given covariate c, the predicted mean abundance of a feature g from the decoder d is:

$$\hat{x}_g^d = Softmax(f_{known}^d(c) + f_{unknown}^d(z)) + \exp(l)$$

Where l is the library size factor. Repeating this for all $d \in \{1, \dots, D\}$ yields a distribution over predicted feature levels, which can be used to calculate confidence intervals and perform statistical tests.

Hypothesis testing: When comparing feature levels under covariate conditions (e.g., treatment vs. control), users can compute the difference in predicted expression across all decoder instances:

$$\Delta_g^d = \hat{x}_g^d(A) - \hat{x}_g^d(B)$$

The distribution of $\{\hat{x}_g^d\}_{d=1}^{D}$approximates the variability in the estimated effect. This can be used to perform hypothesis testing, derive empirical p-values, or assess the robustness of differential expression estimates.

Default hyperparameters

The centra building block in DeepDive (which is stacked to construct encoder, decoder, or adversary networks) is a fully connected block that applies:

$$output = Dropout\left(LeakyReLU\left(BatchNorm1d\big(Linear(Input)\big)\right)\right)$$

Unknown encoder
The encoder maps input counts x into a q-dimensional latent representation consists of N stacked building blocks, where N is a hyperparameter (defaults: N = 2, input dimensions = number of features and 128, output dimensions = 128 and 64, dropout = 0.1). The output of the final building block is projected to the mean vector q_m and the variance vector q_v using linear layers, each with a Z-dimensional (default = 32) output. Finally, the latent variable is sampled using the reparameterization trick on the mean and variance vectors.

Known embeddings
For each covariate c, an embedding is learned using a single linear layer mapping that maps unique levels in the input to a Z-dimensional (default = 32) output. During training, the embedding is regularized by adding noise sampled from a normal distribution given a sigma (default: 0.2). All known embeddings are added to generate a unified known latent variable.

Decoders
The decoder reconstructs chromatin accessibility from latent variables. Decoders are created for both the known and unknown latent variables N stacked building blocks (defaults to the reverse of the unknown encoder). The known and unknown latent variables are decoded to logits, then summed and rescaled to the original feature space.

Adversarial networks
Adversarial networks are applied to each known covariate embedding and each consists of a single build block layer with a Z-dimensional input (default = 32), dropout (default = 0.3) and M-dimensional output (default = 16). This output is projected to logits (c-dimensional for classification, where c is the number of categories) or values (for regression).
